# Classification of Mindfulness Meditation and Its Impact on Neural Measures in the Clinical Population

**DOI:** 10.3389/fpsyg.2022.891004

**Published:** 2022-06-10

**Authors:** Sze Ting Joanna Ngan, Pak Wing Calvin Cheng

**Affiliations:** Department of Psychiatry, The University of Hong Kong, Pokfulam, Hong Kong SAR, China

**Keywords:** mental illness, mindfulness meditation (MM), psychopathology, cognitive neuroscience, classification

## Abstract

Different forms of mindfulness meditation are increasingly integrated in the clinical practice in the last three decades. Previous studies have identified changes in the neurophysiology and neurochemistry of the brain resulting from different mindfulness meditation practices in the general population. However, research on neural correlates of different types of meditation, particularly on the clinical outcomes, is still very sparse. Therefore, the aim of this article is to review the neural impact of mindfulness meditation interventions on different mental disorders via the classification of main components of mindfulness meditation. The clearer classification of mindfulness meditation may inform future clinical practice and research directions.

## Introduction

### What Is Mindfulness Meditation

The introduction of mindfulness meditation (MM)-based interventions is growing in popularity. This may be contributed by its versatility to reach clinical and non-clinical groups, the diversity that provides an alternative to treatment-resistant patients, and its adherence to familiar activities such as yoga and breathing exercises that enhance engagement and adoption of mindfulness meditation-based intervention in our daily lives. Interventions of MM have been shown to reduce stress and improve one’s subjective wellbeing in different age groups and ethnic backgrounds (Turakitwanakan et al., 2013; Black et al., 2015; Ghane et al., 2018; Zollars et al., 2019; Liu et al., 2020; Chen et al., 2021). Due to its positive effect in enhancing individuals’ wellbeing and resilience, the practice of MM attracts research on the underlying mechanism of its effects in the mental health setting.

Mindfulness is the foundational attentional stance implicated in different schools of Buddhist meditation tradition, such as Theravada tradition, Vajrayana tradition, Zen, and Vipassana mediation (Hart, 2011). The word “mindfulness” is translated from the Buddhism Pali’s term “sati,” which means to be mindful and aware of the experience by refining attention and action with a calm and untrained mind (Peacock, 2014). Mindfulness meditation is an umbrella term of the family of meditational practice that describes the action of focusing one’s attention in the present, with the attitude of being non-judgmental, open, and curious of the experience (Bishop et al., 2004).

### Types of Mindfulness Meditation

Various forms of meditation are found in different populations, religions, and cultures. Particularly, there are different types of MM developed specifically for the clinical population. Since the 1990s, Kabat-Zinn developed a secular intervention known as mindfulness-based stress reduction (MBSR) suited for the general population and for those suffering from chronic pain and stress symptoms (Kabat-Zinn, 2003). It was then brought to the clinical population where Segel later modified and developed mindfulness-based cognitive therapy (MBCT) to target specifically clinically depressed patients (Morgan, 2003). The idea of mindfulness is also integrated into other forms of behavioral interventions such as dialectical behavioral therapy developed for borderline personality disorder, and acceptance and commitment therapy (ACT) has been effective in patients with chronic pain, depression, obsessive-compulsive disorder, psychosis, and those under palliative care (Linehan, 1993; O’Hayer et al., 2018; Zhao et al., 2021). MM is adopted to broader mental health populations, such as substance use, anxiety disorder, and eating disorder (Evans et al., 2008; Kristeller and Wolever, 2011; Bowen et al., 2014).

### Main Techniques of Mindfulness Meditation

The different types of MM interventions in practice and in clinical research make it difficult to bring consistent prediction of the effects of MM on psychopathology. However, there are consistencies in the aim of some MM interventions such as MBSR and MBCT, which are commonly found to relieve negative symptoms by enhancing ways to cope with negative thoughts and emotions. In theory, the primary basis of the mindfulness-based interventions consists of two main techniques, namely, focused attention (FA) and open monitoring (OM). During the MM interventions, the instructor will guide practitioners to attend to a specific object (FA) such as bringing awareness to their breathing (Kabat-Zinn, 2003). Meanwhile, practitioners are encouraged to notice if their mind wandered to a task-irrelevant object, i.e., thinking about the to-do grocery list. When noticing the distraction, it is warmly reminded to shift attention back to the self-related processing such as the sensation of breathing and to try and sustain the attention of the experience, i.e., air coming in and out from the nostril. FA enables the enhancement of top-down selective and maintenance of attention against distractors (Fujino et al., 2018). This strengthens the regulation of emotion by reducing sensitivity to emotional distractors, and hence, attending to the attentional focus may help to relieve the perceived intensity of physical and psychological symptoms (Zeidan et al., 2012; Guendelman et al., 2017). On the other hand, OM is the technique of being non-reactively aware of the moment-to-moment thoughts and feelings (Hölzel et al., 2011). It is suggested that FA and OM are gradual processes where FA sets the foundation for mental stability to further achieve OM (Zhang et al., 2019). Instead of avoiding and suppressing emotions, OM promotes the attitude of acceptance. This approach facilitates a more objective and accurate perception of experience and thus helps to further enhance mindfulness and maintain a good mood (Craig, 2002).

### Components of Mindfulness Meditation

Aforementioned, the usual procedures of MM are integrated from a complex and interrelated range of ideas, attitudes, and cognitive processing that may explain the insufficient scientific consensus on the underlying mechanism of MM. Yet, there are core components that are related to improving coping and symptoms of patients (see Table 1). In this section, we described the core components of MM in the cognitive scope to facilitate the understanding of the neural effects of MM on mental illness which are discussed later.

**TABLE 1:** The description of mindfulness meditation (MM) components and neural mechanisms.

MM components	Description	Neural mechanism
Present-centered awareness	Selective attention to present moment sensory and perceptual experience	Salience network: bilateral anterior insular, dorsal ACC
Meta-awareness	Monitor the process of consciousness, mind-wandering, orientate attention back to present moment	Default mode network: posterior cingulate cortex, medial PFC, precuneus and inferior parietal cortices
Non-reactive self-related processing	Non-judgmental, accepting and curious attitude to view self-related perceptual and cognitive process	Narrative focus (cognitive interpretation of mental processes): ventral mPFC, dorsal mPFC, left lateral PFC, middle temporal and angular gyri Experiential focus (attend to present-moment sensory thoughts, feelings and sensations): ventral PFC, dorsolateral PFC, right insular, SII and inferior parietal lobule

*ACC, anterior cingulate cortex; PFC, prefrontal cortex; mPFC, medial prefrontal cortex.*

#### Present-Centered Awareness

Present-centered awareness describes the regulation and temporal structure of attention to the here-and-now feeling, thoughts, and sensations. It is the “aware” and “focus” stage of MM. The practice of MM identifies the prioritization of selectively attending to present moment sensory and perceptual experience (i.e., breathing) (Lutz et al., 2008). The present-centered awareness also requires maintaining focus back at the present moment upon mind-wandering to distractors, which is the “focus” stage. Neuroimaging studies have suggested that the anterior cingulate cortex (ACC) requires top-down regulation that allocates attentional resources by being “aware” of the present moment in disregard of distractor to “focus” on the present (Bush et al., 2000). In relation to the anterior insula, the ACC works as a network to shift between different brain activities to facilitate cognitive control (Menon and Uddin, 2010). MM recruits the salience network where activations were observed in bilateral anterior insular and dorsal ACC implicated in the emotions, conscious perception of bodily response, and present moment awareness (Seeley et al., 2007; Craig and Craig, 2009; Singer et al., 2009). Supportive evidence emerges from functional magnetic resonance imaging (fMRI) studies that greater activation in ACC was found in meditators while practicing present-centered awareness as compared with controls (Hölzel et al., 2007; Gard et al., 2012). In the clinical population, greater awareness of the present moment is evident in improving mood disorders symptoms by enhancing attentional regulation (Brown et al., 2011). Through MM, the training on present-centered awareness may thus be targeted to improve mental illnesses that relate to attentional deficit such as attention deficit hyperactivity disorder (ADHD) and bipolar disorder (Maalouf et al., 2010; Passarotti et al., 2010).

#### Meta-Awareness

Meta-awareness refers to the cognitive capacity to monitor the process of consciousness that meta-awareness of the inevitable stage of mind-wandering during MM facilitates attentional shift back to the task (Smallwood et al., 2007; Mrazek et al., 2012). The practice of meta-awareness requires attention control to continuously adjust one’s attentional focus, which needs response inhibition (Brefczynski-Lewis et al., 2007). The training of meta-awareness helps to reduce mind-wandering (Franklin et al., 2017). Robust activation was found in the default mode network (DMN) during mind-wandering, including the posterior cingulate cortex (PCC), medial prefrontal cortex (mPFC), and inferior parietal cortices (Broyd et al., 2009). These regions are activated during the resting state, which is coherent to internal mental state processes such as self-referential processing, thinking about the past, or imagining the future (Buckner et al., 2008). Research has shown that DMN activities are negatively related to mental health outcomes and attentional demanding task performance (Grimm et al., 2009; Brewer et al., 2011; Fernández-Corcuera et al., 2013; Hamilton et al., 2015). Correspondingly, the DMN is implicated in explaining the underlying mechanism of symptoms as ruminating on the past and worrying about the future are the negative thinking patterns commonly found in individuals with depression, anxiety, substance abuse, and eating disorders (Colvin et al., 2021). In other words, the enhanced meta-awareness via reducing mind-wandering may help to ameliorate the severity symptoms, hence improving wellbeing. In this study, we explored changes related to the process of mind-wandering to understand the impact of meta-awareness on mental illnesses.

#### Non-reactive Self-Related Processing

Non-reactive self-related processing refers to the non-judgmental, accepting, and curious attitude to view the perceptual and cognitive process during MM (Kabat-Zinn, 2003). Self-awareness is a core cognitive process interpreting the relationship between internal and external stimuli to self, and the dual-mode of self-reference suggests that distinct forms of self-focus have differentiated neural mechanisms (Farb et al., 2007). There is a tendency of default bias during resting state to narrative focus that describes the cognitive interpretation of mental processes, recruiting midline PFC (ventral and dorsal mPFC) and linguistic-semantic network (left lateral PFC, middle temporal, and angular gyri) (Mason et al., 2007). In contrast, experiential focus describes the inhibition of cognitive interpretation to attend to present-moment sensory thoughts, feelings, and sensations, recruiting right lateralized network (ventral and dorsolateral PFC) and right insular, SII, and inferior parietal lobule (Gusnard et al., 2001).

Non-reactive processing can be achieved through dereification and acceptance. Dereification describes the practitioner acts as a separate entity from their thoughts. It can also be understood as “psychological distancing” to reduce experiential fusion so that the practitioner can experience and interpret mental processes in a more objective and non-judgmental sense (Bernstein et al., 2015). Dereification can be viewed in the dual mode of self-reference as the shift from narrative focus to experiential focus (Farb et al., 2007). In addition, the acceptance stance is to enhance greater accuracy in perceiving mental contents instead of suppressing the content of mental experience (Dixon et al., 2020). As patients with mood disorders were found to be more active in the DMN than controls that are related to poor mental health, this non-reactive attitude targets to combat the automatic constraints to turn to negative valence during mind-wandering (Broyd et al., 2009). Both techniques of dereification and acceptance promote more mental flexibility which is lacking in patients with mood disorders (Gutierrez et al., 2015).

As different studies investigate the changes MM has to the brain with different measures and components, inconsistent findings were found. This study is going to explore how the different components mentioned above impact different mental illnesses neurologically. The understanding of the underlying neural effects of MM with greater clarity helps to provide a clearer prediction as to how components of MM impact on different mental illnesses and, hence, provide directions for future research and development of clinical practice.

## Methods

### Search Strategy and Inclusion and Exclusion Criteria

A quantitative review search of PubMed, ScienceDirect, PsycInfo, and The Cochrane Library was performed for research articles. In the electronic databases, we searched for studies that included the terms “mindfulness meditation,” “mental illness,” “psychopathology,” “patients,” “meta-awareness,” “mindful attention,” “present-centered awareness,” “acceptance,” “non-reactive,” “brain changes,” “neural correlates,” and “neural mechanism” up until February 2022. The selection criteria included research articles with clinical data and symptoms of mental illness. Studies of all sample sizes were analyzed. Studies were excluded if they were dissertations, reviews, or not published in English. The search resulted in 1,030 records. Excluding studies that did not include the mental health population, 12 studies were included in this review (see Table 2).

**TABLE 2:** Neurological changes in the brain associated with mindfulness meditation.

Article	Age	Clinical population	Sample size	Study design	Outcome
**1. Present-centered awareness**					
Zhao et al., 2019	32.6	Generalised anxiety disorder	37	Repeated measures pre-post 8-weeks MBCT Resting-state fMRI	MBCT: increased ACC and insula functional connectivity related to anxiety improvements
Goldin and Gross, 2010	35.2	Social anxiety disorder	14	Repeated measures Pre-post 8-weeks MBSR React to negative self-beliefs under fMRI Employ attention deployment techniques Breath-focused Distraction-focused (count backwards)	Breath-focused: reduced amygdala activation, increased cuneus and middle occipital related to improved self-esteem, anxiety and depression symptoms
Westbrook et al., 2013	45	Addiction (smoking)	47	Repeated measures React to smoking and neutral pictures fMRI Employ attention deployment techniques (a) Mindful attention (non- judgmental) Look at the picture with no techniques	Mindful attention: reduced functional connectivity between subgenual ACC and the bilateral insula, related to reduce cigarette craving
Schoenberg et al., 2014	36.7	Attention deficit/hyperactivity disorder	44	Randomised controlled trial 12 weeks MBCT (*N* = 24) vs. wait-list control (*N* = 20) Completed Go/No-Go task with ERP	MBCT: increased P3 ERPs amplitudes, activities related to the inhibitory gating circuit
Sibalis et al., 2019	13	Attention deficit/hyperactivity disorder	56	Randomised controlled trial 20 sessions mindfulness-based martial arts treatment: including FA and body scan (*N* = 34) vs. wait-list control (N-22) Completed Go/ No-Go task with EEG	MM: increased EEG beta with oscillation frequency of 12 to 30 Hz and reduced EEG theta with frequency of 4 to 7 Hz, reflecting more concentration and less unfocused thought, respectively
**2. Meta-awareness**					
Chou et al., 2022	37.5	Bipolar disorder	44	Two group repeated measures Bipolar disorder (*N* = 22) vs. healthy control (*N* = 22) 12 weeks MBCT vs. supportive psychotherapy Resting-state fMRI	MBCT: subjects with Bipolar disorder showed negatively correlated dlPFC- PCC activity, related to heightened self-report meta-awareness, rated on the Five Facet Mindfulness Questionnaire scale.
Tang et al., 2013	21.5	Addiction (Smoking)	50	Randomised controlled trial Smokers (*N* = 27) vs. non-smoker (*N* = 33) 2 weeks mindfulness meditation IBMT vs. relaxation training Resting-state fMRI	IBMT: increased activity ACC and PFC, linked to self-control. Reduced activity in PCC, related to reduce craving and cessation of smoking. Maintained reduced smoking after 4 weeks.
Cernasov et al., 2019	35.2	Depression (Anhedonia)	11	Repeated measures Pre-post 15 weeks MBCT fMRI Resting-state fMRI	MBCT: decreased in DMN connectivity between the mPFC and precuneus, correlated to reduced Beck’s Depression Inventory scores
Wells et al., 2013	74	Mild cognitive impairment	14	Randomised controlled trial pilot 8 weeks MBSR (*N* = 9) vs. TAU (*N* = 5) Resting-state fMRI	MBSR: Increased functional connectivity between the PCC and bilateral mPFC and left hippocampus compared to controls. Showed trends of less bilateral hippocampal volume atrophy than control participants.
**3. Non-reactive self-related processing**					
Hölzel et al., 2013	37.9	Generalised anxiety disorder	26	Randomised controlled trial 8 weeks MBSR (*N* = 15) vs. stress management education (*N* = 11) Label angry and neutral face under fMRI	MBSR: increased functional connectivity between amygdala and PFC regions, negatively correlated to Beck Anxiety Inventory scores
King et al., 2016	31	Post-traumatic stress disorder	23	Randomised controlled trial 16 weeks mindfulness-based intervention (*N* = 14) vs. present-centered group (N=9) Resting-state fMRI	MM: increased DMN (PCC seed) connectivity to dlPFC regions within the CEN, correlated to improvement in avoidant and hyperarousal symptoms
Smallwood et al., 2016	46	Comorbid chronic pain and addiction	12	Pilot study Chronic-pain focused ACT (*N* = 6) vs. health education (*N* = 6) At rest and painful stimulation during Resting-state fMRI	ACT: reduced activation in middle frontal gyrus, inferior parietal lobule, insula, ACC, PCC, and superior temporal gyrus, areas related to pain responsiveness. Higher connectivity in amPFC – vmPFC and PCC and insular.

*MBCT, mindfulness-based cognitive therapy; MBSR, mindfulness-based stress reduction; IBMT, integrative body-mind training; ACT, acceptance commitment therapy; MM, mindfulness meditation; TAU, treatment as usual; ACC, anterior cingulate cortex; dlPFC, dorsal lateral prefrontal cortex; mPFC, medial prefrontal cortex; amPFC, anterior medial prefrontal cortex; vmPFC, ventral medial prefrontal cortex; PCC, posterior cingulate cortex; DMN, default-mode network; CEN, central executive network.*

## Discussion

Despite the beneficial effects on wellbeing observed in previous literature, most studies investigate the underlying mechanism of MM by studying the general cognitive impact. There are existing studies targeting on the general neural mechanism on MM (Hölzel et al., 2011; Van Der Velden and Roepstorff, 2015). However, very limited studies provide data on the underlying neural mechanism of MM in the clinical population. To our knowledge, no existing studies learn about the neural impact of the specific components of MM in the mental health population. In this study, in the review of the studies on the specific effect of MM components that induce brain changes, we have identified differentiated mechanisms that bring similar or different impacts on the clinical population.

### Neural Effects of Present-Centered Awareness on Mental Illnesses

Neuroimaging research has proposed that present-centered awareness facilitates attentional regulation in different mental health populations. MBCT appeared to reduce anxiety symptoms significantly in a waiting-list control trial (Zhao et al., 2019). Adult patients with generalized anxiety disorder (GAD) had reduced activation in the bilateral insula with increased functional connectivity to ACC, which was associated with the reduced Hamilton Anxiety Rating Scale scores after 8 weeks of MBCT. The reduced activation in bilateral insula is implicated in the heightened awareness of present emotions and perceptions under the salience network that helps to relieve anxiety symptoms (Friedel et al., 2015; Mooneyham et al., 2017). The ameliorated anxiety scores may be explained by the attentional focus on present-moment experience where maladaptive patterns of thinking and behaviors can be regulated (Chambers et al., 2009). Consistent findings have shown that similar MM practice reduced social anxiety symptoms in patients with social anxiety disorder (SAD) by better visual attentional regulation of negative self-beliefs, reflected by greater MBSR-related activation in cuneus and middle occipital brain regions (Goldin and Gross, 2010). This provides evidence that the strengthened awareness of the present thoughts and emotions helps to regulate one’s emotions, a crucial factor in the recovery process (Young et al., 2019).

The practice of present-centered awareness was also beneficial to enhance attentional regulation in treatment-seeking smokers. Adult subjects who were asked to react to smoking-related pictures had reduced craving to smoking cue upon deploying mindful attention training as compared with controls with no attentional deployment techniques used (Westbrook et al., 2013). A psychophysiological interaction was found in the representation of the effect of mindful attention in the decoupling between subgenual ACC and other craving-related processing such as insula and ventral striatum. The decoupling may suggest that mindful attention strengthens attentional control to present-moment and refrains from engaging in craving-related.

Similar neural effects in the salience network were identified in understanding the impact of MM on ADHD. Schoenberg et al. (2014) revealed that adults with ADHD improved bio-regulation skills and reduced impulsivity symptoms, coincided with enhanced P3 ERPs amplitudes, a component of the inhibitory gating circuit after 12 weeks of MCBT training in present-moment practice and body scan. Similar findings also extended to youths with ADHD that a significant increase in active attention engaging more concentration and less unfocused thought was found in youths performing Go/No-Go tasks. This was reflected by the lower theta/beta ratio after 20 weeks of mindfulness treatment programs where no difference was found in controls (Sibalis et al., 2019).

Studies exploring the neural correlates of present-centered awareness with different measuring modalities including resting-state fMRI, fMRI, and ERP have consistently suggested the significant role of the salience network in regulating attention and improving mood. The particular practice in the MM component of present-centered awareness has provided beneficial effects to patients with anxiety and attentional symptoms. However, studies in the review were mostly conducted with the adult populations, which can be difficult to be generalized to other age groups. The different measurement methods, i.e., evaluating the mind at the resting state as compared with the active processing when reacting to stimuli, can be hard to make comparisons in terms of the specific impact of present-centered awareness on patients.

### Neural Effects of Meta-Awareness on Mental Illnesses

Aberrant DMN activities have been commonly found in different mental illnesses, with their negative impact on self-control, attention to task, and emotional regulation (Broyd et al., 2009). MM seems to have exerted an effect in modifying atypical DMN connectivity in patients with bipolar disorder (BD) that mirrors the healthy controls (Chou et al., 2022). At baseline, patients with BD showed hyperactivation in the dorsal lateral PFC (dlPFC), an area within the mind-wandering-related DMN network, as opposed to the anticorrelated activity between PCC and dlPFC in healthy controls while performing attentional tasks. Upon MBCT training, the atypical dlPFC connectivity was modified as akin to the healthy group which coincided with the heightened meta-awareness, and this change was not found in the control group. In other words, MM seems to enhance attentional regulation in BD.

Further neuroscience research along the same lines comes from Tang et al.’s (2013) study. It highlights the unconscious change of MM to reduced smoking. Reduced PCC activities and increased ACC–PFC connectivity were found to be correlated with reduced smoking in heavy smokers after integrative body-mind training that composes of MM elements, i.e., meta-awareness. The reduced PCC activities reflected reduced resting-state activities that were related to reduced craving and smoking intensity. Specifically, subjects were recruited with the aim of stress reduction, without the intention of quitting smoking. This study provides evidence of the unconscious role of MM in enhancing self-control in smokers.

A similar finding was observed in patients with significant anhedonia that the improved depression symptoms were correlated with reduced connectivity in the DMN, between the medial PFC (mPFC) and the precuneus after 15 weeks of MBCT training (Cernasov et al., 2019). Higher functional connectivity between mPFC and precuneus is implicated in the negative sense of self and self-esteem interpretation usually found in depressive patients (Rolls, 2016). Therefore, the reduced connectivity in this study may suggest that mood improvement may be due to less rumination and negative self-belief that help to explain the improved Beck Depression Inventory scores in patients.

Despite the DMN is marked as a biomarker of its detrimental effect on mental health, the resting-state DMN can be also used as a non-invasive biomarker to evaluate the effect of MM on the clinical population. In a pilot study, Wells et al. (2013) have shown that patients with mild cognitive impairment (MCI) had enhanced DMN connectivity in the hippocampus, mPFC, and PCC after MBSR training as compared with controls. Additionally, reduced hippocampal atrophy was identified more than controls. The altered brain changes suggested that MBSR helps with reserving cognitive resources from the development of MCI. However, factors such as more social engagement were not accounted for the effect; hence, the interpretation of the effect of MM should be of caution. It is further explained that the enhanced connectivity within the DMN stabilizes activities in the DMN, hence deactivating the DMN more adequately when the task demand increases (De Marco et al., 2018). In alignment with previous studies, modified activities in the DMN facilitate mood regulation and task concentration (Whitfield-Gabrieli and Ford, 2012).

Studies exploring the neural impacts of meta-awareness were more consistent in measuring the rest-state activity with the resting-state fMRI. The enhanced meta-awareness has been shown to be implicated in the altered DMN network in patients with BD, addiction, depression, and MCI. Different studies have reflected the role of meta-awareness with different depths and scopes in the DMN network, i.e., Chou and colleagues’ study measured the changed connectivity between PCC and dlPFC, while Tang and colleagues’ study compared the changes in the ACC and PFC connectivity and the activity level in the PCC. The differentiated identification of areas as a seed for neuroimaging may make a precise comparison of the neural changes hard, and thus, the interpretation of the precise changes in the DMN connectivity should be of caution.

### Neural Effects of Non-reactive Self-Relating Processing on Mental Illnesses

A reversed PFC and amygdala connectivity was found in patients with GAD after MBSR. The post-treatment-enhanced connectivity between PFC and amygdala was negatively correlated with Beck Anxiety Inventory Scores (Hölzel et al., 2013). It is explained that the recruitment of more PFC activities is related to engaging in more active monitoring of affective states instead of downregulating or avoiding them, providing evidence for the role of acceptance in improving anxiety symptoms.

The training on the non-reactive self-related processing is also evident in the study of King et al. (2016). An enhanced DMN-central executive network connectivity was revealed in patients with posttraumatic stress disorder (PTSD) after 16 weeks of mindfulness-based exposure therapy but not psychoeducation controls. Particularly, the altered connectivity coincided with the improved avoidant and hyperarousal symptoms in the MM group. This finding provides evidence that the non-reactive and accepting stance to review internal processes promotes less avoidance to emotional experience and may be beneficial to patients with PTSD, enhancing treatment effectiveness. However, no significant difference was observed in the overall PTSD symptoms. The non-significant change in PTSD symptoms may be explained by the focus of acceptance value alone, which may not be targeted to the general PTSD symptoms but more focused on the cognitive and coping mechanism that requires further examination to exert effects on the overall symptoms of PTSD.

The application of ACT is suggested to enhance one’s psychological flexibility by accepting the experience which aligns with the acceptance component of MM (Hayes et al., 2004). This is speculated in the study of patients with comorbid chronic pain and opioid addiction (Smallwood et al., 2016). Patients completed 8 sessions of ACT, and pain symptoms were alleviated reflected by lower activation in the regions related to pain processing, that is, insula, ACC, and PCC, but not in the health education controls. This study did not provide evidence regarding the impact of the core ACT elements, i.e., acceptance and mindfulness on pain processing. However, the increased connectivity pairing of anterior mPFC, ventral mPFC and PCC, right posterior insula during the resting state found in the ACT group may provide support to a stronger self-referential process and a greater awareness to own body’s physiological state that parallels with the lowered perceived pain in patients.

Finally, the non-reactive attitude of self-relating processing was observed with differentiating connectivity in different regions of the brain but with a similar underlying mechanism. This component exerts effects more on the coping mechanism than the overall symptoms. The DMN is involved in the self-relating process, while the non-reactive element brings in interactive regions to impact on the clinical population. Under fMRI, heightened connectivity between PFC and amygdala was reflected in the GAD population during active tasks (Hölzel et al., 2013). In addition, at the resting state, an enhanced DMN-CEN connectivity resulted in patients with PTSD, while increased DMN-insular coupling was found in patients with comorbid chronic pain and opioid addiction (King et al., 2016; Smallwood et al., 2016). The positive connectivity highlights the enhanced role of processing the affective and emotional experience instead of suppressing it, a crucial representation of an accepting attitude of MM. The studies reviewed do not seem to provide evidence of dereification shown by the shift of narrative focus-related regions to experiential focus-related regions in the mental health population. Future studies may explore the dynamics of the aforementioned areas to understand the role and impact of dereification in MM.

### Limitation of Existing Studies and Future Directions

The main limitation of the studies in MM in this review is the absence of a homogenous methodology. Despite resting-state fMRI being a common measure in studying the neural changes of MM, the variation in applying different regions as seeds for DMN can make neural correlates interpretation difficult. In addition, the study methodology to investigate the underlying mechanism of MM can be arbitrary, i.e., inference, instead of introducing measures targeting on different facets of MM, i.e., Five Facet Mindfulness Questionnaire scale to study the neural correlates. Also, the sample size of the studies is relatively small (*N* < 30) (Goldin and Gross, 2010; Hölzel et al., 2013; Wells et al., 2013; King et al., 2016; Smallwood et al., 2016; Cernasov et al., 2019), and the interpretation of the analysis is thus to be cautioned which may not be representative to the whole mental health population. As mentioned earlier, there are existing studies exploring the neural effects of MM, but less to none investigate the neural mechanism according to the components of MM. The challenge of measuring the neural effects of MM may then be contributed by the lack of consensus upon the constitutes of MM. Future studies are suggested to establish a framework of MM components so that studies on the neural effect of MM can dive into the specific impact of each component for in-depth analysis systematically. The clear identification of the neural correlates of each component can then help to provide more evidence-based alternatives and short-term acute support to the clinical population.

## Conclusion

The brain changes identified based on the different MM components have helped to elucidate the specific neural mechanism of treatment effect under each component. Although it is not the focus of this review, the cognitive impact of each component is not distinctively different. However, through reviewing the related neural mechanism of each component, the differentiated functions are better understood. In summary of the review of previous studies, present-moment awareness concerns more on the external attentional regulation that recruits the salience network. The training of meta-awareness emphasizes on the interoceptive regulation of the whole consciousness process that involves the DMN. Finally, non-reactive self-related processing stresses on the attitude of viewing experience concerns of the DMN-central executive network connectivity. These findings have helped to deepen the understanding of the underlying neural mechanism of MM, hence highlighting the need for developing a common framework of MM components for future research in exploring the effects of MM with greater consensus and consistencies in the findings.

## Author Contributions

JN and CC prepared the full manuscript. Both authors contributed to the article and approved the submitted version.

## Conflict of Interest

The authors declare that the research was conducted in the absence of any commercial or financial relationships that could be construed as a potential conflict of interest. The handling editor JG declared a shared affiliation with the author(s) at the time of review.

## Publisher’s Note

All claims expressed in this article are solely those of the authors and do not necessarily represent those of their affiliated organizations, or those of the publisher, the editors and the reviewers. Any product that may be evaluated in this article, or claim that may be made by its manufacturer, is not guaranteed or endorsed by the publisher.
